# Optimization of overlap extension PCR for efficient transgene construction

**DOI:** 10.1016/j.mex.2019.12.001

**Published:** 2019-12-04

**Authors:** Roland S. Hilgarth, Thomas M. Lanigan

**Affiliations:** a Division of Rheumatology, Department of Internal Medicine, University of Michigan, Ann Arbor, MI, 48109, United States; b Vector Core, Biomedical Research Core Facilities, University of Michigan, Ann Arbor, MI, 48109, United States

**Keywords:** Overlap extension PCR, Subcloning, Restriction enzyme free gene splicing

## Abstract

PCR is a powerful tool for generating specific fragments of DNA that can be used to create gene variations or tagged expression constructs. Overlap extension PCR is a valuable technique that is commonly used for cloning large complex fragments, making edits to cloned genes or fusing two gene elements together. After difficulties in utilizing this technique following existing methods, we developed an optimized protocol. To accomplish this, three significant changes were made; 1) touchdown PCR cycling parameters were used to eliminate the need for optimizing PCR cycling conditions, 2) the high-fidelity, high-processivity Q5 DNA polymerase was used to improve full-length amplification quality, and 3) a reduced amount of primer in the final PCR amplification step decreased non-specific amplimers. This modified protocol results in consistent generation of gene fusion products, with little to no background and enhanced efficiency of the transgene construction process.

## Graphical abstract

**Figure:**
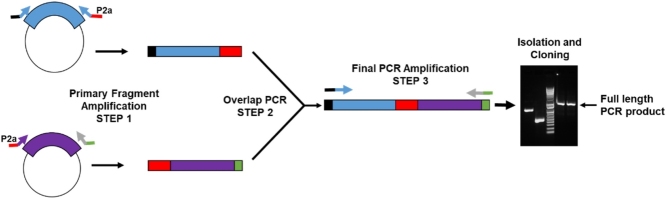

**Specification Table**

**Table:** 

Subject Area:	Biochemistry, Genetics and Molecular Biology
More specific subject area:	PCR gene construction
Protocol name:	Design and Construction of P2A Peptide-Linked Multicistronic Vectors; Generation of P2A Linked Multicistronic Cassettes by Recombinant PCR.
Reagents/tools:	1 X TAE Buffer
	6 X DNA Loading Buffer
	Gel red
	Q5 DNA polymerase (New England Biolabs)
	10 mM dNTP
	Agarose
	Molecular grade water
	DNA electrophoresis chamber
	UV light box
	PCR machine
Experimental design:	To develop a reliable, easy to follow protocol for overlap extension PCR that requires little to no optimization. To accomplish this, we focused on the generation of P2A gene fusion as described by Szmczak et al. [1] and explored several reaction parameters in order to find the optimal conditions of each one. We utilized touchdown PCR cycling conditions throughout the protocol to eliminate the need to optimize the PCR conditions [2]. The effect of DNA polymerase was examined by comparing commonly used Phusion DNA polymerase to Q5 DNA polymerase. Both polymerases are high fidelity but Q5 also has high processivity of DNA synthesis. Lastly, the amount of primer was varied in the final PCR amplification step from the standard 10 μM down to 1 μM.
Trial registration:	N/A
Ethics:	N/A

**Value of the Protocol**

**Table:** 

• Method requires little to no optimization time. • Simple and easy to follow protocol. • The method allows for the easy construction of P2A gene fusion constructs. • Works efficiently for any type of overlap extension PCR manipulation.

## Description of protocol

Overlap extension PCR was originally developed as a method to introduce mutations into transgenes [[3], [4], [5], [6]]. It has since been developed and utilized to generate gene chimeras and more recently been described to be used in the generation of seamless P2A fusion constructs [1,7]. In this paper, the identification of key steps that allow for reliable success of P2A fusion gene construction by overlap extension PCR were tested. The result is an optimized protocol for the generation of P2A fusion genes that relies on the use of a high fidelity DNA polymerase with high processivity, touchdown PCR in all PCR steps, and decreased primer concentration in the final P2A gene fusion PCR amplification step.

**Major equipment and supplies for PCR:**

• PCR thermal cycler (Eppendorf mastercycler or equivalent)
• PCR tubes
• Sterile 1.5 mL Eppendorf style microcentrifuge tubes
• Benchtop microcentrifuge
• Agarose gel electrophoresis apparatus
• Axygen or other gel documentation system
• Adjustable micropipettors (0.1–1000 μL)
• Aerosol-resistant micropipette tips (10–1000 μL)

**Reagents for PCR reactions**

• Phusion DNA polymerase (New England Biolabs, USA)
• Q5 DNA polymerase (New England Biolabs, USA)
• 10 mM dNTP mix (Promega, USA)
• 10X TAE buffer
• Agarose gel
• Primers (IDT, USA or equivalent).

**Reagents for gel cleanup and ligation**

• Monarch® DNA gel Extraction Kit (New England Biolabs, USA)
• Sterile 1.5 mL Eppendorf microfuge tubes
• Restriction enzymes
• T4 DNA ligase (New England Biolabs, USA).
• Nanodrop (ThermoFisher)

## Method details

**Fig. 1:**
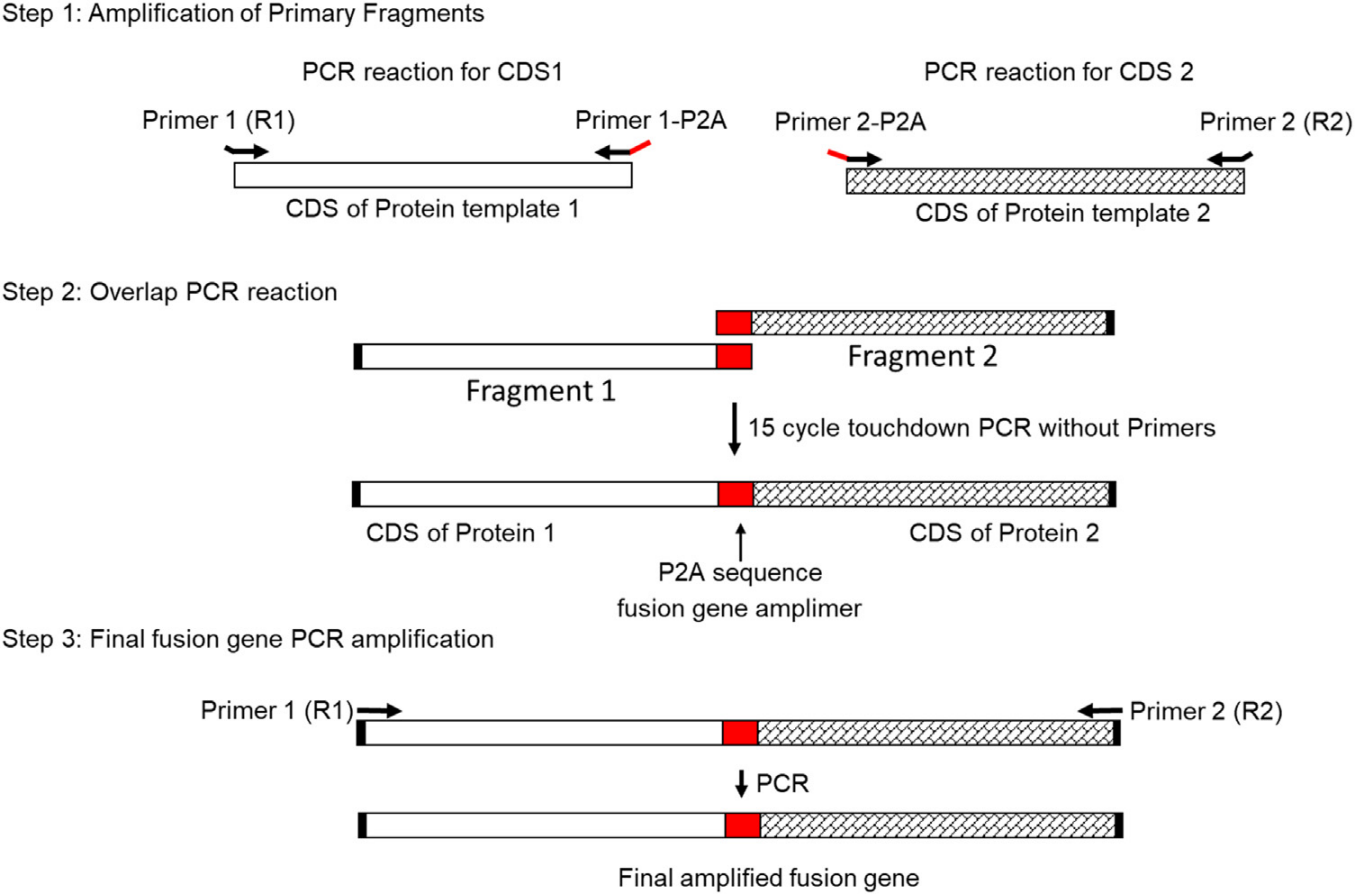
Graphical overview of the generation of P2A linked expression cassette by overlap PCR. Overlap PCR generation of P2A linked expression cassettes is a 3 step process. Step 1 (top panel) uses touchdown PCR to generate PCR fragments that contain a 36 bp P2A sequence overlap (red portion of primers 1-P2A and 2-P2A) at the 3′ end of the Fragment 1 and the 5′ end of the Fragment 2. Restriction enzyme sites are also included in R1 and R2 primers (black bent arrows). Step 2 (middle panel) uses Fragment 1 and Fragment 2 to generate a full length P2A coding sequence (red boxes) fusion using overlap PCR. Step 3 PCR amplifies the full length P2A containing product. The final product has restriction enzyme sites at both ends, with 2 in-frame coding sequences and a P2A site separating them.

In this protocol, we use overlap extension PCR to construct a fusion protein separated by a P2A peptide cleavage site that will allow for separation of the two polypeptides upon expression in the cell [8] (Fig. 1). The coding sequence (CDS) for protein 1 and protein 2 are PCR amplified from expression plasmids and the P2A site will be incorporated through the primer design.

**Fig. 2:**
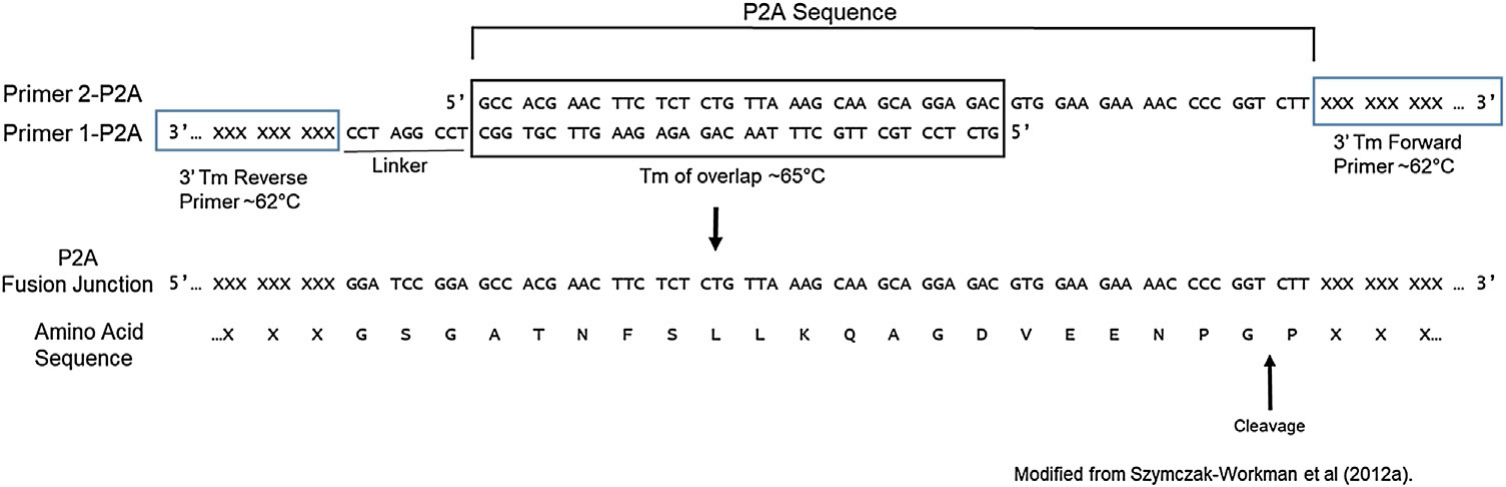
Schematic of the primer design for the addition of P2A peptide sequence for overlap extension PCR. Both primers shown (1-P2A and 2-P2A) have P2A sequence at their 5′ends that are complementary to each other (top panel). The P2A sequence overlap region is boxed and has a Tm of ∼65 °C. Both primers also have CDS specific sequences at the 3′ end (blue boxes). A small linker region is also included between the first CDS and the P2A site (primer 1-P2A). The CDS specific sequences for each primer has a Tm of ∼62 °C. The resulting fusion PCR contains CDS 1 followed by an in frame P2A site and CDS 2 (bottom panel). The peptide sequence and P2A cleavage site are shown.

To design this construct, ensure that the first open reading frame 1) has a Kozak sequence before the initiating methionine (ATG) [9], 2) does not contain a stop codon and 3) is in frame with the P2A expression cassette and second open reading frame. The only stop codon will be at the end of the second open reading frame. An important feature of overlapping PCR is to ensure that primers 1-P2A (reverse primer to amplify CDS1) and 2-P2A (forward primer to amplify CDS2) have overlapping sequences on their 5′ends (Fig.2). This overlapping sequence is incorporated into the PCR products in Step 1 and is crucial to the primer extension in Step 2 (Fig. 1).

All primers should have an annealing temperature of approximately 62 °C so that the fragment generation and final amplification PCR may be done using the same touchdown PCR program with only an elongation step time adjustment [2]. Note that the 62 °C annealing temperature is calculated only from the gene target specific portion of the P2A primers (1-P2A and 2-P2A) in Step 1, and does not include the extended P2A sequence (Fig. 2). Modifying the PCR parameters to touchdown PCR and using the Q5 polymerase greatly reduces time and reagents in amplifying products while maintaining high quality product. Restriction enzyme sites are included in the 5′ ends of the R1 and R2 primers to allow for subcloning of the final product (Fig. 1).

### Amplification of primary fragments (Step 1)

The first amplification generates PCR products with overlapping sequences for the overlapping extension in Step 2. In order to eliminate the need for PCR optimization, we used touchdown PCR in all of our amplification programs. PCR reactions* are carried out in a volume of 50 μL and all reactions were assembled on ice.

**Table:** 

PCR reaction for CDS 1:	PCR reaction for CDS 2:
10 μL 5X Q5 reaction buffer	10 μL 5X Q5 reaction buffer
1 μL 10 mM dNTP	1 μL 10 mM dNTP
3 μL 10 μM primer mix: R1 and 1-P2A	3 μL 10 μM primer mix: R2 and 2-P2A
10 ng of DNA template for protein 1	10 ng of DNA template for protein 2
X μL molecular grade water	X μL molecular grade water
Up to 50 μL using molecular grade water	Up to 50 μL using molecular grade water

*Note: If GC enhancer is needed in the reaction decrease the water by amount by 10 μL and add 10 μL of Q5 GC enhancer. Additionally, in the generation of the primary fragment (Step 1), an alternate high fidelity DNA polymerase, such as Phusion DNA polymerase, may be used.

Thermocycling conditions for the primary PCR reactions are as follows:

94 °C 30 s 1 cycle

94 °C 15 s, 67 °C (−0.5 °C/cycle) 15 s, 72 °C 30 s/kb for 17 cycles

94 °C 15 s, 59 °C 15 s, 72 °C 30 s/kb for 23 cycles

72 °C 2 min 1 cycle.

Hold 10 °C

**Fig. 3:**
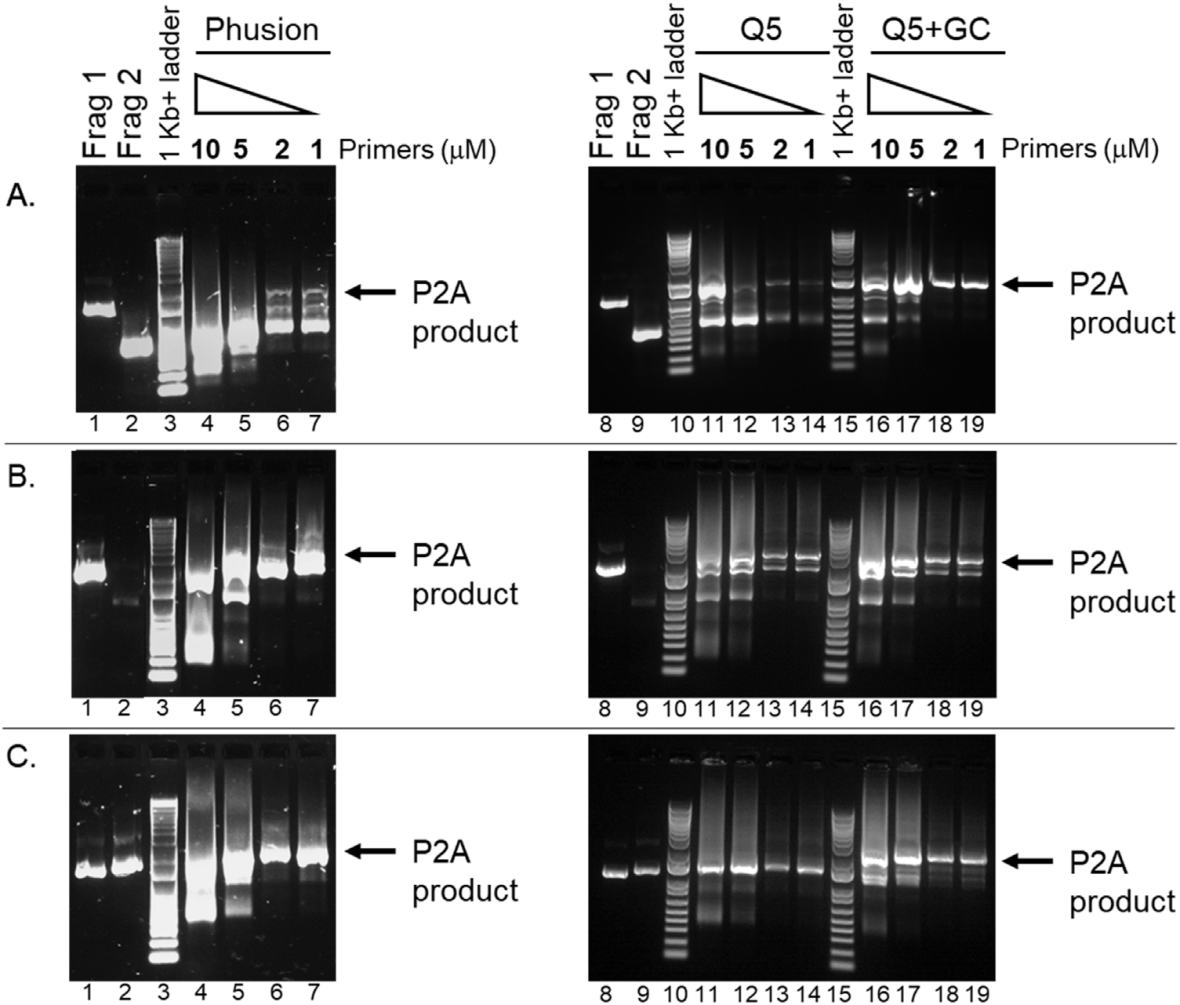
Effect of Polymerase choice and primer concentration on final P2A fusion PCR amplification step. A, B, and C are three different P2A gene fusion constructs (see Table 1). The left panels utilized Phusion DNA polymerase which resulted in inconsistent amplification of the final P2A fusion products. The right panels utilized Q5 DNA polymerase and Q5 DNA polymerase with GC enhancer. The Q5 PCR reactions resulted in consistent amplification of the P2A fusion products (lanes 11–14 and 16–19). Across all PCR conditions, the decrease of primer concentrations from 10 mM to a range of 1−2 mM resulted in cleaner amplification of the P2A fusion products (lanes 13, 14, 18, 19).

Analyze 30 μL of PCR reaction by gel electrophoresis on a 1 % agarose TAE gel containing 1X Gel Red (Biotium) and run at 10 V/cm for 30 min. Visualize amplimers under ultraviolet light (Fig. 3) and excise PCR products of the predicted sizes for Fragment 1 and Fragment 2 using a clean razor blade. Place individual gel slices in separate 1.5 mL Eppendorf tubes and recover the DNA using the Monarch® DNA gel extraction kit following the manufacturer’s instructions. Elute DNA off the column using 30 μL of elution buffer.

Purified primary PCR products should then be quantified using a Nanodrop® and all purified DNA should be stored at −20 °C until needed for the overlap PCR reaction (Step2).

### Overlap PCR reaction (Step 2)

The purpose of the Overlap PCR reaction is to generate the full-length fusion gene containing the P2A site from the two primary fragments generated in Step 1. This PCR reaction does not use any primers and relies on the overlapping sequences generated in Step 1 for primer extension. It is important that both primary fragments be present in the PCR reaction in equimolar amounts. We generally use ∼100 ng/1 kb fragment in the PCR reaction. Calculation of molar ratios may be done using a biomath calculator such as NEBioCalculator (https://nebiocalculator.neb.com/#!/ligation). The overlap PCR reactions were carried out in a volume of 50 μL. P2A fusions with fluorophores can be GC rich. To streamline the process, we recommend setting up two PCR reactions, one with GC enhancer and one without, and do the same for step 3: final fusion gene PCR amplification. The PCR reactions using Q5 DNA polymerase with and without GC enhancer are listed below.

**Table:** 

Overlap Q5 Reaction:	Overlap Q5 + GC enhancer Reaction:
10 μL 5X Q5 reaction buffer	10 μL 5X Q5 reaction buffer
2 μL 10 mM dNTP	10 μL 5X Q5 GC enhancer
X μL Primary Fragment 1 (∼100 ng/Kb)	2 μL 10 mM dNTP
X μL Primary Fragment 2 (∼100 ng/Kb)	X μL Primary Fragment 1 (∼100 ng/Kb)
0.5 μL Q5 DNA polymerase	X μL Primary Fragment 2 (∼100 ng/Kb)
Up to 50 μL using molecular grade water	0.5 μL Q5 DNA polymerase
	Up to 50 μL using molecular grade water

Thermocycling conditions for the overlap PCR reaction are as follows:

94 °C 30 s 1 cycle

94 °C 15 s, 72 °C (−0.5 °C/cycle) 30 s/kb for 9 cycles

94 °C 15 s, 67.5 °C (−0.5 °C/cycle) 15 s, 72 °C 30 s/kb for 5 cycles

72 °C for 2 min 1 cycle.

Hold 10 °C

PCR cleanup is not needed for the final amplification in Step 3. PCR products can be stored at −20 °C.

### Final fusion gene PCR amplification (Step 3)

The purpose of the final PCR step is to amplify the full-length P2A fusion product for DNA isolation and subcloning. There are two key factors in this step. The first is to utilize a high processivity, high fidelity DNA polymerase such as Q5 or PfuUltra®. The second key factor is to use low concentrations of primer 1 (R1) and primer 2 (R2) from Step 1 (amplification of primary fragments). Lowering the concentration of primers in the reaction greatly enhances the yield and lowers the presence of non-specific bands. The final PCR reactions are as follows:

**Table:** 

Final Fusion Reaction:	Final Fusion + GC enhancer Reaction:
10 μL 5X Q5 reaction buffer	10 μL 5X Q5 reaction buffer
2 μL 10 mM dNTP	10 μL 5X Q5 GC enhancer
4 μL Step 2 PCR reaction	2 μL 10 mM dNTP
1 μL primer 1 (R1) from Step 1 (1–2.5 μM)	4 μL Step 2 PCR reaction
1 μL primer 2 (R2) from Step 1 (1–2.5 μM)	1 μL primer 1 (R1) from Step 1 (1–2.5 μM)
0.5 μL Q5 DNA polymerase	1 μL primer 2 (R2) from Step 1 (1–2.5 μM)
Up to 50 μL using molecular grade water	0.5 μL Q5 DNA polymerase
	Up to 50 μL using molecular grade water

Thermocycling conditions for the final fusion PCR reaction are as follows:

94 °C 30 s 1 cycle

94 °C 15 s, 67 °C (−0.5 °C/cycle) 15 s, 72 °C 30 s/kb for 17 cycles

94 °C 15 s, 59 °C 15 s, 72 °C 30 s/kb for 23 cycles

72 °C 2 min 1 cycle.

Hold 10 °C

Analyze 30 μl of the PCR reaction using gel electrophoresis on a 1 % agarose TAE gel containing 1X gel red and run at 10 V/cm for 30 min. Visualize the DNA bands under ultraviolet light (Fig. 3) and excise amplimers that are the correct size for the full-length fusion gene using a clean razor blade and place in a 1.5 mL Eppendorf tube. Purify the DNA from the agarose gel slice using the Monarch® DNA gel extraction kit following the manufacturer’s instructions and elute in a final volume of 30 μL.

The final PCR product can be subcloned into your expression vector using the restriction sites on the 5′ and 3′ end of the generated fusion gene (from R1 and R2 primers) and then sequenced to verify that there are no PCR errors in the final construct. For the purposes of validating the efficacy of the altered protocol, we subcloned the full-length fragment and selected clones for sequencing.

## Validation

To validate this protocol, we generated and cloned 3 separate P2A containing fusion genes. We examined the effects of primer concentration and different polymerases used to generate the final fusion gene (Step 3) (Fig. 3). The amplification of the final fusion gene can be affected by primer concentration and DNA polymerase choice. From our experience, reducing the primer amount to 1–2 μM in the final PCR reaction (Step 3) significantly improves the amplification of the desired PCR product in all conditions. Additionally, the use of a high processivity DNA polymerase appears to increase the quality of desired amplimer produced. We also believe it is worth setting up PCR reactions with and without GC enhancer for the overlap PCR reaction (Step 2) and the final fusion gene amplification (Step 3) due to the possibility of GC rich repeat areas. The efficiency of this protocol has further been confirmed by the number of clones verified with the correct full-length sequence (Table 1).

**Table 1:** Validation of Correct Gene Fusion Generation by Overlap Extension PCR.

	Step1		Step 2 & 3		
Construct	Size of Fragment 1 (Kb)	Size of Fragment 2 (Kb)	Size of Final Gene Fusion (Kb)	Number of Clones Sequenced	Number Correct
Gene fusion A	0.95	0.44	1.4	5	4
Gene fusion B	1.6	0.78	2.38	5	3
Gene fusion C	1.0	1.1	2.1	5	5

## Conclusion

Due to difficulties in getting consistent results using overlap extension PCR following existing methods, we developed an optimize protocol that provides reliable, consistent results each time. This protocol works efficiently for any type of overlap extension PCR manipulation, including 1) mutagenesis by insertion, deletion or point mutations, 2) inserting epitope tags or P2A sites, and 3) splicing sequences together. The optimization of the protocol comes from changes in primer amounts, DNA polymerase and cycling conditions.

## Declaration of Competing Interest

The authors declare that they have no known competing financial interests or personal relationships that could have appeared to influence the work reported in this paper.

## References

[1] Szymczak-Workman A.L., Vignali K.M., Vignali D.A. (2012). Generation of 2A-linked multicistronic cassettes by recombinant PCR. Cold Spring Harb. Protoc..

[2] Don R.H., Cox P.T., Wainwright B.J., Baker K., Mattick J.S. (1991). ‘Touchdown’ PCR to circumvent spurious priming during gene amplification. Nucleic Acids Res..

[3] Horton R.M., Ho S.N., Pullen J.K., Hunt H.D., Cai Z., Pease L.R. (1993). Gene splicing by overlap extension. Methods Enzymol..

[4] Nelson M.D., Fitch D.H. (2011). Overlap extension PCR: an efficient method for transgene construction. Methods Mol. Biol..

[5] Sambrook J., Russell D.W. (2006). Site-specific mutagenesis by overlap extension. Cold Spring Harb. Protoc..

[6] Forloni M., Liu A.Y., Wajapeyee N. (2018). Creating insertions or deletions using overlap extension polymerase chain reaction (PCR) mutagenesis. Cold Spring Harb. Protoc..

[7] Szymczak-Workman A.L., Vignali K.M., Vignali D.A. (2012). Design and construction of 2A peptide-linked multicistronic vectors. Cold Spring Harb. Protoc..

[8] Liu Z., Chen O., Wall J.B.J., Zheng M., Zhou Y., Wang L., Ruth Vaseghi H., Qian L., Liu J. (2017). Systematic comparison of 2A peptides for cloning multi-genes in a polycistronic vector. Sci. Rep..

[9] Kozak M. (1987). An analysis of 5'-noncoding sequences from 699 vertebrate messenger RNAs. Nucleic Acids Res..

